# Some Remarkable Rheological and Conducting Properties of Hybrid PVC Thermoreversible Gels/Organogels

**DOI:** 10.3390/gels8090557

**Published:** 2022-09-01

**Authors:** Dominique Collin, Ganesh Viswanatha-Pillai, Andreas Vargas Jentzsch, Odile Gavat, Emilie Moulin, Nicolas Giuseppone, Jean-Michel Guenet

**Affiliations:** Institut Charles Sadron, CNRS—Université de Strasbourg, 23 rue du Loess, 67034 Strasbourg CEDEX 02, France

**Keywords:** PVC thermoreversible gels, organogels, hybrid materials, rheology, conductivity

## Abstract

We report on investigations into the rheological properties of organogels prepared from triarylamine trisamide (TATA) and oligo phenylene vinylene (OPVOH) molecules in binary organogel gels and in ternary thermoreversible networks with poly vinyl chloride (PVC). In the case of OPVOH, we show that the modulus of the ternary gel is simply the sum of the modulus of each binary gel, corresponding to the so-called Voigt upper limit. In contrast, TATA/PVC ternary gels generally exceed the Voigt upper limit. In an attempt to account for this unexpected outcome, we hypothesized that a de-solvation process might occur in the PVC fibrils that possibly originates in the propensity of TATA molecules to form molecular compounds with the solvent. Finally, the conducting properties of TATA/solvent organogels and TAT/PVC/solvent reversible networks were measured. It was found that they strongly depend on the solvent type but are not significantly altered when PVC is present. Therefore, PVC gels can be made conducive by incorporating TATA fibers.

## 1. Introduction

Functional materials are usually obtained from an appropriate mixture of several components that eventually imparts the required property. The oldest example mastered by humankind is unquestionably the making of metallic alloys [1]. In the 20th century, new materials were developed from polymers that also contain additives to improve and/or alter their basic properties [2]. 

More recently, a wealth of self-assembling systems has been synthesized that often possess interesting properties, such as opto-electronic properties, conductivity, recognition capability and so on [3,4,5,6,7,8,9]. These systems form fibrillar morphologies in many solvents, which results in the production of organogels. These are reminiscent of polymer thermoreversible gels, which are also arrays of fibrillar structures. Making hybrid materials by merging these two types of networks seems legitimate and has recently been successfully developed [10,11]. Indeed, they allow one to impart new functional properties to widely-used polymers without any additional chemistry. This paper focuses on two organogel systems: those prepared from oligo phenylene vinylidene (OPV), which display opto-electronic properties—i.e., they change color at the SOL-GEL transition; and those prepared from triaryl trisamide (TATA), which possesses electric conductivity (see materials and methods).

Various remarkable properties observed in hybrid gels prepared from PVC are presented in this paper, especially rheological properties. They highlight the interest of further investigating these systems—and especially those prepared from PVC, an important commodity polymer [12]—for potential applications.

## 2. Results and Discussion

We first present the rheological properties in order to highlight the unusual, unexpected outcomes obtained from hybrid PVC/organogelator gels. Then, we present results concerning the conductivity of hybrids gels imparted by the presence of a conducting organogelator—namely, triarylamine trisamide—dispersed within a PVC matrix.

### 2.1. Rheological Properties

All the gels studied here were characterized by G′ > > G″ in the explored frequency range. They therefore complied with the accepted definition of gels from the rheological point of view [13,14,15]. They further complied with the definition given for thermoreversible gels; namely, gels made of fibrillar entities and exhibiting first-order transitions for their formation and melting temperatures [9].

#### 2.1.1. PVC/Oligo Phenylene Vinylene Systems

**Figure 1:**
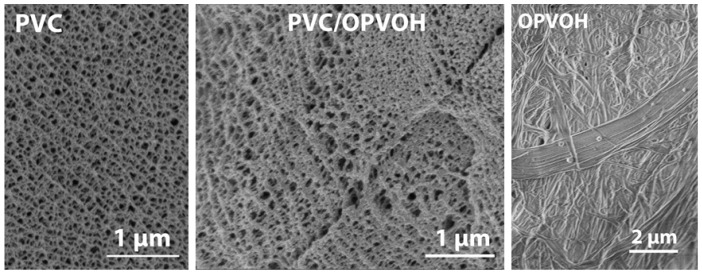
Photomontage from scanning electron microscopy images of PVC gels (*C_PVC_v* = 0.4 × 10^−2^ g/cm^3^) (**left**), OPVOH gels (*C_OPVOH_* = 0.5 × 10^−2^ g/cm^3^) (**right**) and the hybrid system with the same concentrations (**center**).

Hybrid PVC/OPVOH hybrid gels have been previously studied in bromobenzene, a solvent where both entities form fibrillar networks [16]. As can be seen in Figure 1, the morphology of the hybrid gel looks like knitwear and, most importantly, differs from the morphologies of its components. One might wonder whether the OPVOH fibrils grew alongside the PVC fibrils, which would be reminiscent of composite materials. 

**Figure 2:**
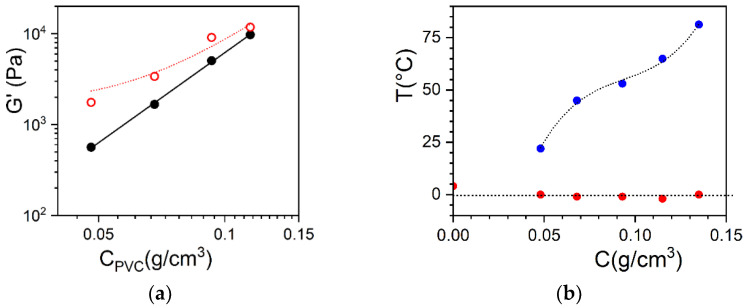
(**a**) Elastic modulus G′ as a function of PVC concentration; black dots = PVC binary system, red open circles = hybrid gels where the OPVOH concentration is kept constant (*C_OPVOH_* = 0.5 × 10^−2^ g/cm^3^), unlike the PVC concentration. The red line is a fit with Equation (4). (**b**) Temperature-composition formation phase diagram, where the PVC composition was varied while the OPVOH concentration was kept constant at *C_OPVOH_* = 4 × 10^−3^ g/cm^3^. The gelation temperature of PVC was obtained from rheological experiments, while the gelation temperatures of the OPVOH/bromobenzene were obtained using DSC. As can be seen, the PVC network always forms first [16].

The rheological study consisted of measuring the elastic modulus as a function of PVC concentration for a fixed OPVOH concentration of *C_OPVOH_* = 4 × 10^−3^ g/cm^3^ (see Figure 2).

Two models developed for fiber-reinforced materials are usually considered. These models normally represent limiting cases. The first is the Voigt model [17], also designated as the upper limit, where the modulus of the hybrid system is simply the sum of the modulus of each species:

(1) $$G_{hyb}^{\prime }=G_{PVC}^{\prime }+G_{OPVOH}^{\prime },$$

The other limiting case is the Reuss model [18], where the modulus of the hybrid species reads:

(2) $$\frac{1}{G_{hyb}^{\prime }}=\frac{1}{G_{PVC}^{\prime }}+\frac{1}{G_{OPVOH}^{\prime }},$$

The Voigt and Reuss models are not limited to composite materials, as they have been successfully applied to the case of thermoreversible gels prepared from compatible biopolymers [19,20].

We considered the Voigt model for the interpretation of previous results published elsewhere on PVC/OPVOH hybrid gels, as shown in Figure 2 [16]. The variation in the elastic modulus of PVC/bromobenzene gels has been found at T = 10 °C:

(3) $$G_{PVC}^{\prime }=4.62\times 10^{6}C_{PVC}^{3.0}$$

The fit is performed with an allometric type function by using Equations (1) and (3):

(4) $$G_{hyb}^{\prime }=G_{OPVOH}^{\prime }+4.62\times 10^{6}C_{PVC}^{3.0}$$

This yields a theoretical value of $G_{OPVOH}^{\prime }$ = 1090 ± 660 Pa, whereas the shear modulus of the OPVOH organogel measured independently at *C_OPVOH_* = 4 × 10^−3^ g/cm^3^ for the binary system gives $G_{OPVOH}^{\prime }$ = 980 Pa. This value is in good agreement with that obtained from the theoretical fit, which confirms the relevancy of the Voigt model for the present system. The origin of the additivity of the elastic moduli is not presently clear, especially at the molecular level.

That none of the basic components of the gel can be clearly identified, as opposed to the systems presented below, may give some clues. The gel may consist of TATA and PVC (b) fibrils inextricably intertwined; hence, the absence of a clear distinction between either network. It should also be kept in mind that, in the hybrid system, the PVC network forms first in this solvent with a mesh size of about 80–100 nm (see Figure 1). As a result, OPVOH fibrils, which possess cross-sections in the binary gels of about 100–500 nm, are forced to grow within a confined space [16]. Whether this contributes to the present morphology of the hybrid gels and to their rheological properties remains an open issue.

#### 2.1.2. PVC/Triarylamine Trisamide Systems

**Figure 3:**
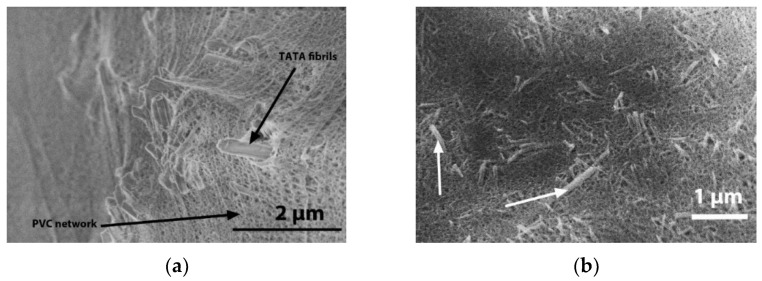
(**a**) SEM image of a hybrid PVC/TATA/bromobenzene gel. The PVC network and the TATA fibrils are indicated by black arrows. (**b**) SEM image of a hybrid PVC/TATA/tetrachloroethane gel. The PVC network and the TATA fibrils are indicated by white arrows.

Hybrid PVC/TATA gels differ drastically both in terms of morphology and rheological properties. SEM investigations of PVC/TATA/bromobenzene and PVC/TATA/TCE are shown in Figure 3a and 3b, respectively [21,22].

**Figure 4:**
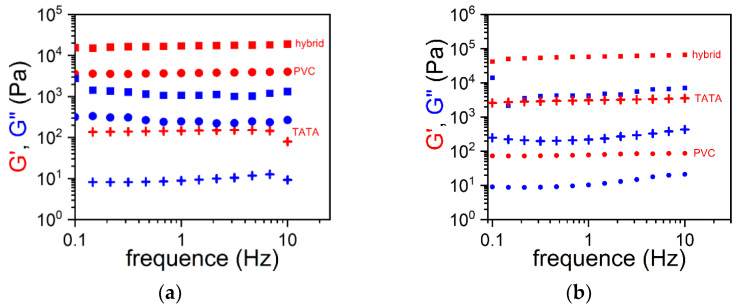
(**a**) Values of the elastic modulus G′ (red symbols) and the loss modulus G” (blue symbols) as a function of the oscillatory frequency for gels prepared in bromobenzene; 🞥 = TATA/bromobenzene organogel (C_TATA_ = 0.5 × 10^−2^ g/cm^3^), ● = PVC/bromobenzene gels (C_PVC_ = 4.8 × 10^−2^ g/cm^3^), ■ = hybrid PVCTATA/bromobenzene gel (C_TATA_ = 0.5 × 10^−2^ g/cm^3^, C_PVC_ = 4.8 × 10^−2^ g/cm^3^. (**b**) Values of G′ (red symbols) and G″ (blue symbols) as a function of the oscillatory frequency for gels prepared in tetrachloroethane; 🞥 = TATA/TCE organogel (C_TATA_ = 1.5 × 10^−2^ g/cm^3^), ● = PVC/TCE gels (C_PVC_ = 4.8 × 10^−2^ g/cm^3^), ■ = hybrid PVCTATA/TCE gel (C_TATA_ = 1.5 × 10^−2^ g/cm^3^, C_PVC_ = 4.8 × 10^−2^ g/cm^3^).

In both systems, the PVC network and the TATA fibrils can be clearly distinguished. The PVC gel mesh size and the TATA fibril cross-sections are nearly the same as those observed in their binary systems. The variations in G′ and G″ for both systems are nearly constant in the explored frequency range, which again confirms the gel status, as does the morphology (Figure 4a,b). New results obtained for PVC/TATA/TCE gels (Figure 4b) confirm those previously obtained for bromobenzene (Figure 4a): the elastic modulus of the hybrid gels was significantly larger than the highest value observed in the binary systems. In other words, the hybrid gels’ moduli exceeded the Voigt upper limit.

Hybrid PVC/TATA gels differ drastically both in terms of morphology and rheological properties. SEM images of PVC/TATA/bromobenzene and PVC/TATA/TCE are shown in Figure 3a and 3b, respectively [21,22]. In both systems, one can clearly distinguish the PVC network and the TATA fibrils. The PVC gel mesh size and the TATA fibril cross-sections are nearly the same as those observed in their binary systems. The variation in the G′ and G″ of both systems are nearly constant in the explored frequency range, which again confirms the gel status, as does the morphology (Figure 3a,b). These new results obtained for PVC/TATA/TCE gels (Figure 3b) confirmed those previously obtained for bromobenzene (Figure 3a): the elastic modulus of the hybrid gels was significantly larger than the highest value observed in the binary systems. In other words, the hybrid gels’ moduli exceeded the Voigt upper limit.

The binary gels prepared from bromobenzene PVC possessed a modulus larger than that of TATA, while the reverse situation was seen for gels in TCE. It has been shown in previous investigations of the gelation mechanism of PVC that molecular compounds can be formed through electrostatic interactions when the solvent possesses some polarization [23]. As a result, the modulus is enhanced due to the creation of junctions. This is the case with bromobenzene, where the bromine atom tends to attract electrons (see Figure 4a). In contrast, TCE is a symmetrical molecule, so electrostatic interaction cannot occur (Figure 4b). Only plasticization of the amorphous domain occurs. This explains in part why the elastic modulus of the PVC gels was higher in bromobenzene than in TCE.

In attempting to account for the unusual behavior of the modulus in the hybrid systems, the equation derived by Jones and Marques [24] for rigid gel exhibiting enthalpic elasticity is worth considering:

(5) $$G_{r}^{\prime }\propto {\mathrm{er}}_{\sigma }^{4}\times {\left(\frac{\varphi_{\mathrm{net}}}{r_{\sigma }^{2}}\right)}^{\left(3+D_{f}\right)/\left(3-D_{f}\right)},$$

where *φ*_net_, r_σ_, e and D_f_ are the fraction of elastic material, the fibrils’ average cross-section, the fibrils’ intrinsic modulus and the fractal dimension of the fibril long axis, respectively.

In the case of PVC, the exponent for the variation of G′ with concentration is close to 3 [23], which gives a fractal dimension of the fibrils’ long axis of about D_f_ = 1.5. If we consider that there are very low amounts of pendant fibrils—i.e., fibrils not participating in the network elasticity—then we can assume that *φ*_net_ ≈ *C_PVC_*, so that Equation (6) reads:

(6) $$G_{r}^{\prime }\propto {\mathrm{er}}_{\sigma }^{4}\times {\left(\frac{C_{PVC}}{r_{\sigma }^{2}}\right)}^{3}\sim \;\frac{e}{r_{\sigma }^{2}}C_{PVC}^{3},$$

**Figure 5:**
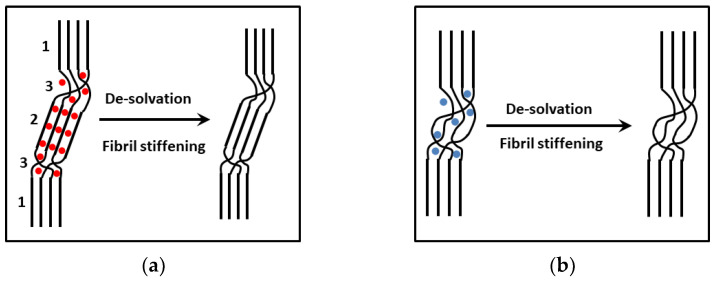
(**a**) Left: sketch of a PVC fibril showing organized sections made up of the highest syndiotactic sequences (1), a solvated section corresponding to the formation of a PVC/solvent molecular compound [23] (2) and the solvated amorphous parts (3); right: after total de-solvation. (**b**) Left: sketch of PVC fibrils where no molecular compounds are formed; only organized sections and the plasticized domains are present. De-solvation occurs in the swollen amorphous domains (right).

To account for the increase in the hybrid gel modulus at constant *C_PVC_*, only two parameters need be considered; specifically, r_σ_ and e. r_σ_ should decrease substantially, which does not seem to be the case in view of the similarities in the cross-sections of the fibrils of either moiety compared to their binary systems. Therefore, it is necessary to contemplate the case where the intrinsic fibrils’ modulus increases. In a previous paper, we hypothesized that the presence of TATA molecules may trigger de-solvation of the amorphous domains by altering the PVC-solvent equilibrium. This may be related to the fact that TATA forms molecular compound with a large variety of solvents, as has been recently shown [25]. This may entail a de-plasticization of the PVC (Figure 5) and, correspondingly, a stiffening of the fibrils, leading to a larger intrinsic modulus and, eventually, a larger elastic modulus, according to Equation (6). Such a de-plasticization effect has already been suggested by Li and McKenna to address an unexpected reinforcement of the mechanical properties of PMMA/graphene oxide compounds [26,27].

It has been established that the gel modulus G′ vs. the swelling ratio S varies as G′ ≈ S^2.72^ (S = V∞/Vo, where Vo is the initial volume and V∞ is the volume after swelling to equilibrium) [23]. Since PVC gel moduli usually vary as G~C^3^, a 15% change in the gel dimension leads to de-swelling of the PVC by about 1.5 and, correspondingly, entails a threefold increase in the modulus. Clearly, a slight change in solvent quality may drastically alter the elastic modulus.

So far, the de-plasticization effect seems to be the best available hypothesis for explaining this unusual rheological behavior. The propensity of TATA molecules to form molecular compounds, as shown recently, is possibly the underlying mechanism responsible for the de-solvation process in the PVC fibrils. TATA molecular arrangements may “absorb” solvent to the detriment of PVC.

**Figure 6:**
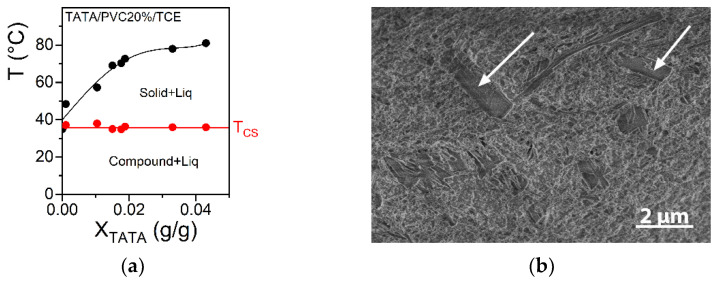
(**a**) Temperature–TATA concentration phase diagram in tetrabromoethane (TBE) as mapped out by DSC at 5 °C/min. A non-variant first-order transition can be seen at T = 40 °C corresponding to the de-solvation of compound C1 into another less solvated compound C2 or a solid phase S. The final melting of the gel was concentration-dependent. The red line is a guide for the eyes. (**b**) cryoSEM micrograph for PVC5%/TATA1.5% in bromobenzene. The TATA fibrils (long ribbons highlighted by arrows) can be clearly distinguished from the surrounding PVC network.

The relation of this effect with the propensity of TATA to form molecular compounds was further backed up by gels prepared in tetrabromoethane (TBE). The temperature–concentration phase diagram displayed in Figure 6a is consistent with the occurrence of a TATA/TBE incongruent-melting molecular compound.

The cryoSEM investigation showed basically the same morphology as that observed in the PVC/TATA/bromobenzene and PVC/TATA/TCE; namely, TATA fibrils dispersed within the PVC matrix (Figure 6b).

**Figure 7:**
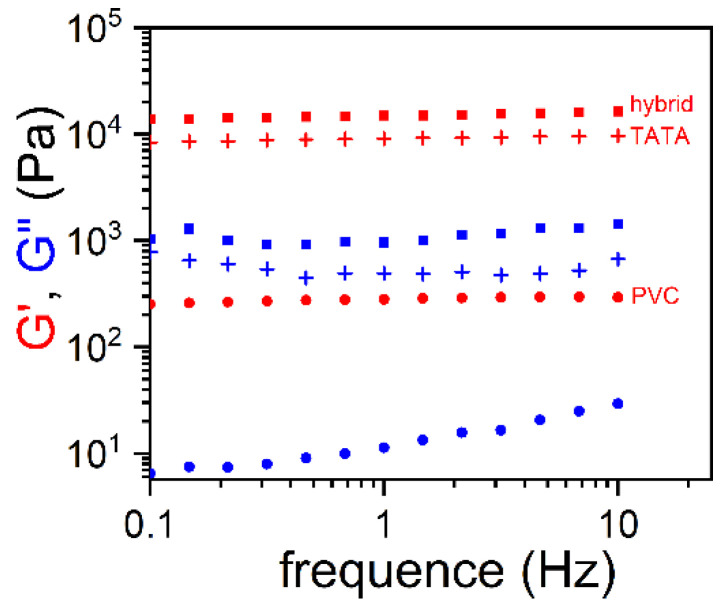
Values of G′ (red symbols) and G” (blue symbols) as a function of the oscillatory frequency for gels prepared in tetrabromoethane; 🞥 = TATA/TBE organogel (C_TATA_ = 1.5 × 10^−2^ g/cm^3^), ● = PVC/TBE gels (C_PVC_ = 4.8 × 10^−2^ g/cm^3^), ■ = hybrid PVCTATA/TBE gel (C_TATA_ = 1.5 × 10^−2^ g/cm^3^, C_PVC_ = 4.8 × 10^−2^ g/cm^3^).

The determination of G′ and G” through oscillatory experiments as a function of frequency again showed that the gel modulus of the ternary system, TATA/PVC/TBE, was higher than the Voigt upper limit (Figure 7).

Therefore, the hybrid gel modulus G′ is larger than the Voigt upper limit in PVC/TATA systems where the TATA organogel forms first and is embedded within the PVC matrix.

### 2.2. Conductivity Properties of PVC/Trisamide Triarylamine Gels in Different Systems

**Figure 8:**
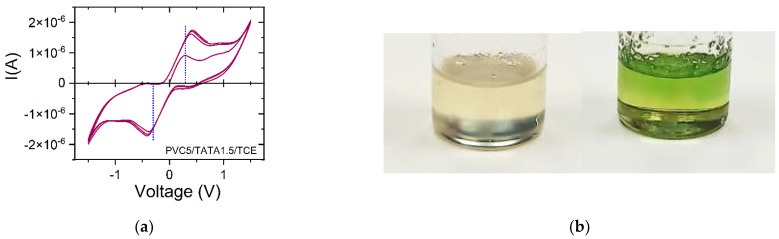
(**a**) Typical voltamogramms obtained at a scanning rate of 100 mV/s, I vs. V, for TATA/solvent and PVC/TATA/solvent samples. The ohmic domain is shown by blue dotted lines. (**b**) Visual presentation of a freshly prepared gel (**left**) and the gel after oxidation (**right**).

These hybrid gels possess interesting conducting properties when trisamide triarylamine (TATA, see Figure 8) molecules are used [28,29,30]. TATA organogels display quite high ohmic conductivity in some solvents, of which is TCE one [28,29,30,31,32]. Making hybrid gels from PVC and TATA allows one to impart conductivity to an otherwise insulating polymer.

A description of the custom-made device used for current versus voltage measurements is given in the Materials and Methods section. Typical voltamogramms with *I* vs. *V* are displayed in Figure 8a for several cycles performed at a rate of 100 mV/s [33]. The conductivity was ohmic in the range from −0.2 V to +0.2 V; i.e., the current varied linearly with the voltage. All the reported values were measured within this range to safely exclude electrochemical processes. From this voltage range, it was possible to derive the ohmic conductivity through *σ*:

(7) $$\sigma \propto \frac{I}{V}\times \frac{l}{A}\;,$$

where *l* is the sample’s thickness and *A* the sample’s surface. The conductivity is expressed in Siemens/m (S/m).

**Figure 9:**
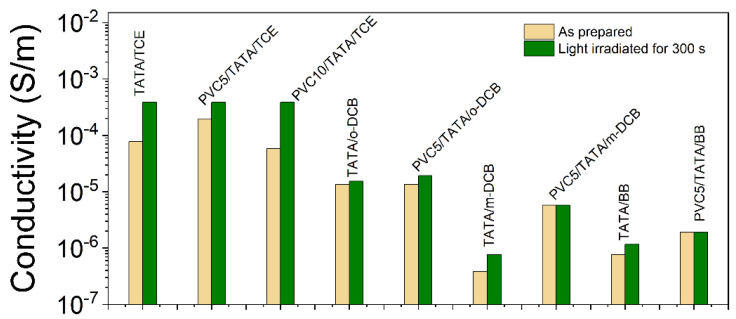
Conductivity in a log scale measured for different samples as indicated. Gray = freshly prepared and green = after 300 s light exposure. In all cases, C_TATA_ = 1.5% (*w*/*w*), C_PVC5_ = 5% (*w*/*w*) and C_PVC10_ = 10% (*w*/*w*). The conductivity of PVC10% in TCE was about 10^−6^ S/m.

The conductivity measurements were systematically carried out on freshly prepared samples, which were later exposed to light irradiation for t = 300 s. Systems displaying conductivity usually change color after irradiation (Figure 8b). TATA binary samples were prepared in four different solvents, TCE, *ortho*-dichlorobenzene (*o*-DCB), *ortho*-dichlorobenzene (*m*-DCB) and bromobenzene (BrBz), at a concentration of C_TATA_ = 0.015 g/cm^3^. The comparison was performed with ternary samples containing 5 to 10% PVC but with the same TATA concentration. The results are displayed in Figure 9 and the values are reported in Table 1.

As can be seen, the highest conductivities were obtained in TCE, as expected from previous work. For the freshly prepared ternary gels, the conductivity of PVC5/TATA/TCE was about the same as the TATA/TCE binary gel and higher than that observed in PVC10/TATA/TCE systems. Conductivity increased significantly in the three systems after exposure to light—by about five times for t = 300 s—reaching almost the same values in the binary and in the ternary systems. Clearly, the PVC matrix had no effect on the TATA conductivity behavior. This further suggests that conductivity can be imparted to PVC gels under this preparation process.

The measured conductivity was much lower in the solvents other than TCE and whether samples were freshly prepared or irradiated had little effect. The highest values were obtained in *o*-DCB, and here, again, conductivity was nearly the same in the ternary systems and in the TATA binary gels. In *m*-DCB, the conductivity was significantly lower—by about one order of magnitude—than in *o*-DCB, which emphasizes the isomer effect. Finally, bromobenzene systems displayed the lowest conductivity.

**Figure 10:**
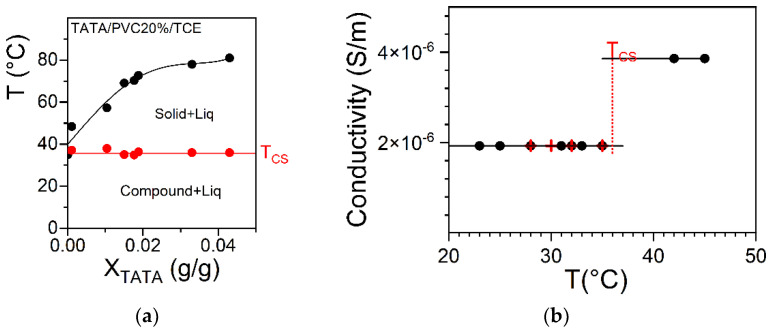
(**a**) Temperature–concentration phase diagram for a fixed PVC concentration (20% *w*/*w*); *T_CS_* is a non-variant event corresponding to the incongruent melting of the TATA/TCE compound [22,25]. (**b**) Conductivity as a function of temperature for hybrid PVC/TATA gels with 20% *w*/*w* PVC concentration; T_CS_ is the transition temperature seen in the T–C phase diagram. Measurements performed upon heating (●) and cooling (🞥).

The variation in the conductivity due to an alteration to the TATA molecular structure within the same sample has been recently reported for the case of hybrid TATA/PVC/TCE gels. For a 20% *w*/*w* PVC composition, the temperature–TATA concentration phase diagram [22] reveals a non-variant first order transition at T = 37 °C (Figure 10a). As recently shown, this corresponds to the incongruent melting of a TATA/TCE molecular compound that transforms into a solid phase [25]. Interestingly, the conductivity displays a jump above T = 37 °C, an effect that is temperature-reversible (Figure 10b). Therefore, the conductivity doubles when the TATA molecular structure changes from a molecular compound to a solid phase. This twofold increase shows that the presence of solvent around the TATA helices is not a prerequisite for triggering their ohmic conductivity.

## 3. Conclusions

The results presented herein again highlight the potential of introducing low-molecular-weight gelators into PVC thermoreversible gels for imparting compelling, unanticipated properties. Rheological investigations revealed a substantial enhancement of the PVC gel modulus, well beyond the Voigt upper limit in the case of TATA molecules. The hypothesis further developed here [21], considering a relation between the propensity of TATA to form molecular compounds with a series of solvents and the de-plasticization of the PVC fibrils, still needs to be thermodynamically understood.

Conductivity measurements show that PVC gels can be made conducive and to reach values close to those measured with the TATA binary gels. This shows that the organogel scaffold is not perturbed by the surrounding PVC matrix. This further underlines the interest of preparing materials from polymers gels and organogels [9,10].

## 4. Materials and Methods

The PVC used in the present study was purchased from Sigma-Aldrich (France) and was used as received. Its characteristics were: Mw = 7.9 × 10^4^ g/mol and the polydispersity index Mw/Mn = 1.87. ^13^C-NMR investigations have the following values for the tetrads: sss = 41% ssi, iss, sis, … = 39% and iii = 19%.

**Figure 11:**
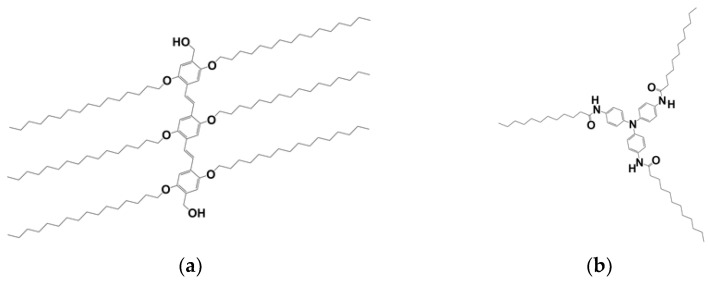
(**a**) OPVOH molecules: the aliphatic arms contain 16 carbon atoms and the terminal groups are OH groups; (**b**) TATA molecules: the aliphatic arms contain 11 carbon atoms.

The organogels were prepared from solutions of oligo vinylene phenylene, designated OPVOH (see Figure 11a), and triarylamine trisamides, designated TATA (Figure 11b). The synthesis and properties of these molecules are extensively described in previous work [6,27,28,29,30,31]. Bromobenzene (BrBz), *ortho*-dichlorobenzene (*o*-DCB), *meta*-dichlorobenzene (*m*-DCB) and 1,1,2,2 tetrachloroethane (TCE) were purchased from Sigma-Aldrich (purity grade 99.5%) and used throughout this study without further purification.

The preparation of the binary gels (PVC/BrBz, OPVOH/BrBz, TATA/BrBz, PVC/TCE, TATA/TCE, TATA/*o*-DCB, PVC/*o*-DCB, TATA/*m*-DCB and PVC/*m*-DCB) and of the hybrid networks (PVC/TATA/BrBz, PVC/TATA/TCE, PVC/TATA/*o*-DCB and PVC/TATA/*m*-DCB) consisted of heating mixtures of the different components up to 120–140 °C until clear, homogeneous solutions were obtained. These solutions were then quenched at low temperatures (usually 0 °C) to produce the gels.

**Rheology**: The real and imaginary parts of the complex shear modulus were measured with a stress-controlled rheometer (Haake, Mars III) operating in the oscillatory mode. The complex moduli as a function of frequency were determined by means of a double Couette cell (DG41, Haake) with a gap of ~400 μm. Approximately 7 cm^3^ of the hot solution was necessary for the experiments. The temperature in the Couette cell was monitored by means of an external bath (Haake F3). For all the shear measurements the stresses applied were lower than σ = 5 × 10^−2^ Pa so as to remain within the linear regime.

The required solutions were prepared by heating mixtures at appropriate temperatures (120–140 °C). After casting these solutions within the Teflon cylindrical cavity and setting the electrodes appropriately, the cell was closed with the upper plate and finally cooled in a fridge at 4 °C for 15 min. Once the gel had set in, conductivity measurements were performed at room temperature at a rate of 100 mV/s.

**Scanning electron microscopy**: Samples were investigated by means of a FEG-cryoSEM from Hitachi (SU8010) at 1 keV and at T = −150 °C. The images were taken with the SE-in lens detector. A piece of gel from the PVC/PVC/TATA/solvent was deposited onto a cryo-holder and then plunged into nitrogen slush in the preparation chamber of a Quorum PT 3010 machine. After transfer into the chamber attached to the microscope, the sample was coated with a thin Pt layer by sputter deposition and finally fractured with a razor blade. Subsequent etching was carried out at appropriate temperatures to gradually reveal the gel morphology.

**Differential scanning calorimetry (DSC)**: The gel formation and melting were investigated using a DSC 8500 from Perkin Elmer. Three heating and cooling rates were used: 5 °C/min, 10 °C/min and 15 °C/min. The range of investigated temperatures was from −20 °C to 150 °C. The temperature–concentration phase diagram was mapped out on the basis of the data obtained at 5 °C/min. Gel samples were prepared separately from homogeneous solutions quenched at 0 °C. About 30 mg of gel was then transferred into stainless steel pans that were hermetically sealed by means of an o-ring.

**Figure 12:**
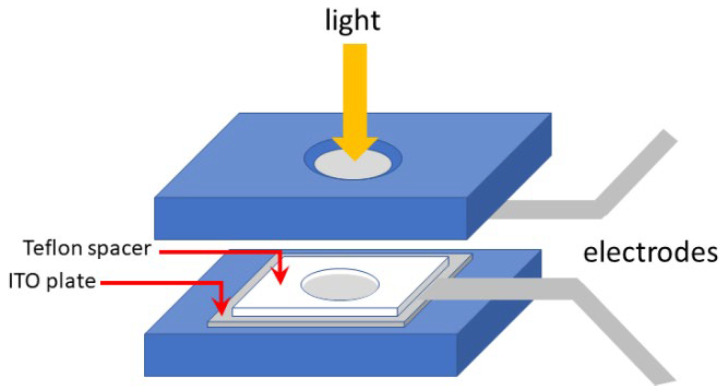
Device used for measuring the conductivity of the different gels. The gel was cast within the hole of the 1 mm thick pacer. The electrodes were placed on each face of the gel before closing the system. Light could be shone onto the sample to oxidize the TATA moiety, as shown.

**Conductimetry**: A cell [32] developed in-house was used (Figure 12). It consisted of two metallic plates separated by a Teflon spacer possessing a cylindrical cavity of known dimensions, into which the hot solution was poured to produce a gel of controlled dimensions. The upper plate possessed a hole that allowed a light to be shone into it for the photo-induced oxidation of the gel sample. The conducting surfaces (electrodes) were made of commercially available transparent ITO plates (ITO glass, 20 × 20 × 1.1 mm, 15 Ω, Lumtec, Taiwan) and glued to the metallic plates. Electric connections were achieved by means of two metal electrodes connected to the two ITO plates. The *I*-*V* curves were measured using a Keithley 2400 Standard Series SMU source meter, which can simultaneously produce current and measure intensity and voltage.

## Figures and Tables

**Table 1:** Values of the conductivity for different binary and ternary gels expressed in Siemens/m.

Sample	As Prepared	After Irradiation
TATA-1.5%/TCE	7.72 × 10^−5^	3.86 × 10^−4^
PVC5/TATA-1.5/TCE	1.93 × 10^−4^	3.86 × 10^−4^
PVC10%/TATA-1.5/TCE	5.79 × 10^−5^	3.86 × 10^−4^
TATA-1.5%/o-DCB	1.35 × 10^−5^	1.54 × 10^−5^
PVC5/TATA-1.5%/o-DCB	1.35 × 10^−5^	1.93 × 10^−5^
TATA-1.5%/m-DCB	3.86 × 10^−7^	7.72 × 10^−7^
PVC5/TATA-1.5%/m-DCB	5.79 × 10^−6^	5.79 × 10^−6^
TATA-1.5%/BrBz	7.72 × 10^−7^	1.16 × 10^−6^
PVC5/TATA-1.5/BrBz	1.93 × 10^−6^	1.93 × 10^−6^

## Data Availability

Row data available on request.
